# The Interaction of Life Course Socioeconomic Status and Leisure Activities on Cognitive Performance in Old Age

**DOI:** 10.1093/geroni/igab046.991

**Published:** 2021-12-17

**Authors:** Rongjun Sun, Zhenmei Zhang

**Affiliations:** 1 Cleveland State University, Cleveland, Ohio, United States; 2 Michigan State University, Department of Sociology, Michigan, United States

## Abstract

While the separate effects of socioeconomic status and engaging in leisure activities on cognition have been well documented, their interaction effect has rarely been examined. After examining life course socioeconomic status (SES) on cognitive impairment in old age, this paper is focused on exploring the interaction effects between life course SES and leisure activities. We use data from the Chinese Longitudinal Healthy Longevity Survey, which covers five waves of interviews of adults aged 65 or older between 2002 and 2014. Cognitive impairment is measured by the Chinese version of Mini-Mental Status Examination. Two sets of variables are used to reflect an older person’s life course SES in childhood and adulthood, respectively. Seven leisure activities are included in this analysis. We adopt the lagged independent variable approach and a Generalized Linear Mixed Model to examine the association between leisure activity and cognitive impairment over time. Results show that there is an independent impact of SES in both childhood and adulthood on cognitive decline in Chinese older population. Furthermore, as the focus of this study, there are substantial interactions between life course SES and engaging in leisure activities with a consistent pattern: those of higher life course SES enjoy extra benefits from engaging in leisure activities. The interactions between life course SES and leisure activities promise a competing approach accounting for cognitive health inequality among older adults.

